# Optimized Rivastigmine Nanoparticles Coated with Eudragit for Intranasal Application to Brain Delivery: Evaluation and Nasal Ciliotoxicity Studies

**DOI:** 10.3390/ma14216291

**Published:** 2021-10-22

**Authors:** Mansi Bhanderi, Jigar Shah, Bapi Gorain, Anroop B. Nair, Shery Jacob, Syed Mohammed Basheeruddin Asdaq, Santosh Fattepur, Abdulhakeem S. Alamri, Walaa F. Alsanie, Majid Alhomrani, Sreeharsha Nagaraja, Md. Khalid Anwer

**Affiliations:** 1 Department of Pharmaceutics, Institute of Pharmacy, Nirma University, Ahmedabad 382481, India; 12mph105@nirmauni.ac.in; 2 Department of Pharmaceutical Sciences and Technology, Birla Institute of Technology Mesra, Ranchi 835215, India; bapi.gn@gmail.com; 3 Department of Pharmaceutical Sciences, College of Clinical Pharmacy, King Faisal University, Al-Ahsa 31982, Saudi Arabia; anair@kfu.edu.sa (A.B.N.); sharsha@kfu.edu.sa (S.N.); 4 Department of Pharmaceutical Sciences, College of Pharmacy, Gulf Medical University, Ajman 4184, United Arab Emirates; sheryjacob6876@gmail.com; 5 Department of Pharmacy Practice, College of Pharmacy, AlMaarefa University, Dariyah, Riyadh 13713, Saudi Arabia; 6 School of Pharmacy, Management and Science University, Seksyen 13, Shah Alam 40100, Malaysia; dr_santosh@msu.edu.my; 7 Department of Clinical Laboratory Sciences, The Faculty of Applied Medical Sciences, Taif University, Taif 26571, Saudi Arabia; a.alamri@tu.edu.sa (A.S.A.); w.alsanie@tu.edu.sa (W.F.A.); m.alhomrani@tu.edu.sa (M.A.); 8 Centre of Biomedical Sciences Research (CBSR), Deanship of Scientific Research, Taif University, Taif 26571, Saudi Arabia; 9 Department of Pharmaceutics, Vidya Siri College of Pharmacy, Off Sarjapura Road, Bangalore 560035, India; 10 Department of Pharmaceutics, College of Pharmacy, Prince Sattam Bin Abdulaziz University, Al-Alkharj 11942, Saudi Arabia; m.anwer@psau.edu.sa

**Keywords:** nanoparticles, rivastigmine, intranasal application, optimization, quality by design

## Abstract

Rivastigmine, a reversible cholinesterase inhibitor, is frequently indicated in the management of demented conditions associated with Alzheimer disease. The major hurdle of delivering this drug through the oral route is its poor bioavailability, which prompted the development of novel delivery approaches for improved efficacy. Due to numerous beneficial properties associated with nanocarriers in the drug delivery system, rivastigmine nanoparticles were fabricated to be administer through the intranasal route. During the development of the nanoparticles, preliminary optimization of processing and formulation parameters was done by the design of an experimental approach. The drug–polymer ratio, stirrer speed, and crosslinking time were fixed as independent variables, to analyze the effect on the entrapment efficiency (% EE) and in vitro drug release of the drug. The formulation (D8) obtained from 2^3^ full factorial designs was further coated using Eudragit EPO to extend the release pattern of the entrapped drug. Furthermore, the 1:1 ratio of core to polymer depicted spherical particle size of ~175 nm, % EE of 64.83%, 97.59% cumulative drug release, and higher flux (40.39 ± 3.52 µg.h/cm^2^). Finally, the intranasal ciliotoxicity study on sheep nasal mucosa revealed that the exposure of developed nanoparticles was similar to the negative control group, while destruction of normal architecture was noticed in the positive control test group. Overall, from the in vitro results it could be summarized that the optimization of nanoparticles’ formulation of rivastigmine for intranasal application would be retained at the application site for a prolonged duration to release the entrapped drug without producing any local toxicity at the mucosal region.

## 1. Introduction

The neurological and neurodevelopmental conditions are the main reason for disability-adjusted life years (DALYs) and the second leading cause of death globally, accounting for 9 million deaths per year. The main contributors of neurological DALYs in 2016 were stroke (42.2%), migraine (16.3%,) dementia (10.4%), meningitis (7.9%), and Epilepsy (5%). Dementia results from a number of diseases and injuries that primarily or secondarily affect the brain. Alzheimer disease (AD) is the most frequent form of dementia and may contribute to 60–70% of cases [1]. The worldwide cases of AD are increasing drastically and are likely to be 74 million by 2030 [2]. The prevalence of this disease is relatively high in Asia and Africa. As the world population is growing older, the socioeconomic consequences of AD are immense and pose a serious challenge for healthcare in modern society. The main reason for the higher costs associated with AD is due to prolonged survival with costly disease-modifying treatment. Presently, for the current 35 million patients with dementia, societal cost is more than $600 billion per year, which is nearly 1% of global Gross Domestic Product [3].

Due to progressive damage or death of neurons during the neurodegenerative disease conditions, such as Parkinson disease, AD, or Huntington disease, researchers are facing the challenge to transport the drugs to the brain at effective concentrations without producing any toxic manifestation [4,5]. Aging is the chief cause of such neurodegeneration and the increasing geriatric population in the near future will further aggravate the present situation [6,7]. Conditions of patients with AD are expressed by the loss of intellectual ability and cognition and impairment of memory due to changes in the morphology of the brain. Prediction of increasing numbers of AD patients in the coming decades has initiated scientists to explore novel deliveries to transport therapeutics directly to the brain [8]. Drug delivery through non-invasive intranasal routes has gained tremendous attention by formulation scientists in the last few decades [9]. Compared to conventional drug delivery systems, nasal drug delivery represents a non-invasive approach with additional advantages such as the rapid onset of action and reduced side effects by a more targeted drug delivery [10]. This route is considered a better alternative for delivering therapeutics to the brain via the olfactory and trigeminal pathways, circumventing the rigid blood–brain barrier that limits the access of actives to the central nervous system while administered using conventional oral or parenteral routes [11].

Rivastigmine is a US FDA-approved natural para-sympathomimetic agent that reversibly inhibits acetylcholinesterase and butyl cholinesterase to treat dementia associated with AD [12]. Such inhibition of cholinesterase increases the brain concentration of acetylcholine to facilitate recovery from memory loss and cognitive deficits due to selective loss of cholinergic neurons in the cerebral cortex, nucleus basalis, and hippocampus [13,14]. However, the oral bioavailability of rivastigmine tartrate is very low, 36%, because of extensive first-pass metabolism and its hydrophilicity [15,16]. Further, this agent is associated with severe gastrointestinal side effects, when administered orally [17]. Thus, there is an urgent requirement to solve the issues with this drug, the low bioavailability, and side effects. Considering the advantages of intranasal administration of therapeutics to directly deliver the drugs to the brain, the present study attempted to deliver this potent agent to the brain using intranasal delivery. The literature signifies that in situ Pluronic F-127 hydrogel constituting Eudragit RL-100 nanoparticles has been previously investigated for the intranasal administration of rivastigmine [18]. Ex vivo studies reported significant permeability enhancement through sheep nasal mucosa in comparison to the drug solution. However, the cumulative percentage of drug released ranged between 60–80% after 24 h and the amount of drug permeated per unit area was rather limited, ranging between 16 × 10^−4^ to 49 × 10^−4^ mg/cm^2^ min after 8 h. In another study, Polysorbate 80-coated poly(n-butyl cyanoacrylate) nanoparticles significantly transported (3.82 fold) rivastigmine to the brain after intravenous administration, when compared to a free drug solution [19]. However, this approach has certain drawbacks such as systemic toxicity, patient non-compliance, and distribution of drug to non-target tissues.

On the contrary, delivering therapeutics using an intranasal route needs special attention for retention at the site with a prolonged-release profile so that the released drug could be transported to the brain [10,20]. Being a popular delivery system for controlled release characteristics, properly designed nanoparticles are known to sustain the release of encapsulated drugs over a longer period from the polymeric matrix [21]. Different polymers have been used to obtain polymer/active ingredient composite systems with rapid, controlled, or targeted delivery. Among the others, PVP [22], zein [23], or PLLA [24] have been utilized to prepare nanoparticles with controllable morphology, encapsulation, release, and improvement of bioavailability.

Further, wide varieties of available components provide the options to choose the desired ingredients favoring mucoadhesion for intranasal application. For example, chitosan, a cationic polymer from a natural origin and its thiolated derivatives have received tremendous attention because of its non-irritant, non-toxic, biocompatible, and biodegradable characteristics along with significant mucoadhesive properties [8,25,26]. Chitosan is reported to disrupt the intercellular tight junctions, hence enhancing the permeability of an epithelium [27]. Similarly, Eudragit^®^ EPO is a cationic terpolymer from the poly(methacrylate) family specifically used as a coating polymer to overcome humidity-related instability of dosage forms and also has good mucoadhesive properties [28,29]. The combination of the repeating methacrylate units within this polymer promotes its solubility under acidic conditions, which is applicable in the design of dosage forms targeted for nasal mucosal region with a pH range typically between 5.5–6.5. Excellent mucoadhesive properties of Eudragit^®^ EPO have been demonstrated using freshly excised sheep nasal mucosa [29]. Due to the inherent clearance mechanism that exists in the nasal cavity, exploitation of mucoadhesive agents along with mucoadhesive coating may extend the time of contact between the drug and the mucus layer, disrupt tight junctions, enhance permeation, and prolong the duration of action, respectively. Therefore, the current investigation aimed to develop mucoadhesive rivastigmine loaded in chitosan and coated with eudragit for intranasal delivery, which could circumvent the first-pass metabolism of drugs and help to achieve a sustained drug release over an extended period. To our knowledge, this is the first study of its type where the rivastigmine was loaded with chitosan and coated with eudragit EPO for the purpose of intranasal delivery. Various formulation and processing parameters were optimized to obtain chitosan nanoparticles having the desired quality. Later, the optimized formulation was coated using Eudragit, to prolong the drug release, and evaluated for in vitro release and permeation study. Finally, the ciliotoxicity study of the coated nanoparticles was done to evaluate the effect on sheep nasal mucosa.

## 2. Materials and Methods

### 2.1. Materials

Rivastigmine tartrate (purity > 98%) was procured from Cadila Healthcare, Mumbai, India. Low-molecular-weight chitosan (50,000 Da; 75–85% deacetylated) was purchased from Sigma Aldrich, Bangalore, India. Eudragit EPO was received from Evonik India, Mumbai, India. Span 80 and glutaraldehyde were obtained from SD Fine Chemicals, Mumbai. Glacial acetic acid was procured from High Purity Lab Chemicals, Mumbai, India. Light and heavy liquid paraffin were obtained from Central Drug House, New Delhi, India.

### 2.2. Preliminary Optimization of Process Parameters for Blank Nanoparticle Preparation

To achieve various desirable properties of the nanoparticles, several preliminary trials were done. First, blank nanoparticles were prepared to select the external phase and the stirrer position. Selection of the external phase was done by experimenting with three different phases, light liquid paraffin, heavy liquid paraffin, and the combination of light and heavy liquid paraffin at a ratio of 1:1. Similarly, to optimize the location of the stirrer, it was set at the top, middle, and bottom positions to check its effect on the formation of nanoparticles.

### 2.3. Optimization of Parameters to Fabricate Drug-Loaded Nanoparticles

To optimize the amount of chitosan, three different batches of nanoparticles (P1, P2, and P3) were prepared using 2%, 3%, and 4% (weight percent) chitosan, respectively. The other components were fixed to fabricate the formulations and to characterize accordingly. Once the percentage of chitosan was optimized during the preliminary process, the amount of drug (batch P4 (150 mg), P5 (100 mg), and P6 (75 mg)) was modified to check the influence of the drug on the formulation characteristics. In a similar process, the volume of span 80 (batch P7 (1%), P8 (2%), and P9 (3%)) in the formulation, volume percentage of crosslinking agent (i.e., glutaraldehyde) (batch P10 (2 mL), P11 (3 mL), and P12 (4 mL)), stirrer speed (batch P13 (1000 rpm), P14 (1500 rpm), and P15 (2000 rpm)), and crosslinking time (batch P16 (2 h), P17 (3 h), and P18 (4 h)) were varied one after another and evaluated to obtain the product having the desired quality.

### 2.4. Full Factorial Design of the Rivastigmine-Loaded Nanoparticles

The above preliminary trials provide us the data to consider the various process and formulation variables for the preparation of the optimization batch. Accordingly, the drug-to-polymer ratio, stirrer speed, and crosslinking time were found to significantly affect the % drug release after 8 h and entrapment efficiency (% EE) of the fabricated nanoparticles. Thus, for the optimization process using the design of experiments’ statistical technique, these three variables were selected as independent variables, and % drug release after 8 h and % EE were selected as dependent variables. A 2^3^ full factorial design (Design Expert^®^ software, version 12, Stat-Ease Inc. Minneapolis, MN, USA) was applied [30] to check the effects of independent variables on dependent variables at two levels demonstrating low and high, respectively (Table 1). The design formulation batches (D1–D8) representing independent variables with coded values and their levels are depicted in Table 1.

### 2.5. Preparation of Nanoparticles

The emulsion crosslinking method was selected for the preparation of mucoadhesive chitosan nanoparticles containing rivastigmine tartrate by a method described previously with minor modifications [31]. Accurately weighed amounts of drug and chitosan were added to 10 mL of prepared acetic acid solution (2% *w/v*) placed in a glass beaker. The dispersion was initially stirred with a glass rod, followed by sonication in a bath sonicator (Trans-o-sonic, D-compact, Mumbai, India) to facilitate the dissolution. The Sonication process continued until chitosan dissolved completely and formed a transparent gel. Meanwhile, the required quantity of light liquid paraffin and Span 80 were taken in another beaker. The mixture was stirred for 10 min using a stirrer (Remi Instruments, Ahmedabad, India) at room temperature. Thereafter, the drug–polymer solution was added dropwise to the external oil phase with continuous stirring at constant rpm for 15 min. Thereafter, glutaraldehyde solution (strength) was added dropwise to the emulsion to facilitate crosslinking and stirring continued for 3–5 h. The suspension was left to stand for 20 min to allow nanoparticles to sediment under the force of gravity. The supernatant was discarded, and the remaining portion constituting nanoparticles with small amounts of oil was separated employing a vacuum filter (NOVA instruments, Mumbai, India). The nanoparticles were then washed 4–5 times using petroleum ether to remove the traces of oil from the surface of nanoparticles and freeze-dried at −60 °C for 24 h. Finally, it was air-dried for 24 h at room temperature (25 ± 1 °C) and stored in a cool and dry place.

### 2.6. In Vitro Release of Rivastigmine from the Designed Batches

The dialysis tube method was used to carry out the in vitro drug release study of the fabricated nanoparticles. Accurately weighed nanoparticles equivalent to 6 mg of drug dispersed in 2 mL of simulated nasal fluid (pH 6.4) were taken in a cellophane dialysis bag (molecular cutoff 12–14 kDa) and placed in the receptor compartment (20 mL of simulated nasal fluid). The temperature of the receiver fluid was maintained at 37 ±  0.5 °C and 100 rpm was set throughout the study [32]. A sample (3 mL) was withdrawn from the receptor compartment at various time intervals and a fresh solution of the same volume was replaced to maintain sink condition. The collected samples were analyzed at 220 nm, and Lambert–Beer’s equation was used for the calculation of % of drug released at different time points. Kinetics and possible mechanisms of drug release from formulations were evaluated by fitting the data into various mathematical models, as described elsewhere [33].

### 2.7. % Entrapment Efficiency and Drug Loading

To determine the % EE of the fabricated formulation, accurately weighed (70 mg) nanoparticles were pulverized in a glass mortar. Then, 50 mg of the crushed nanoparticles were weighed and placed into a flask containing 50 mL of methanol. The flask was shaken using a magnetic stirrer (400 rpm) for 24 h and kept aside for 4 h to solubilize the entrapped drug in the nanoparticles. Filtration of the solution was done using Whatman filter paper (Grade 602 h) to separate the polymers from the supernatant. Finally, the concentration of the drug in the methanol was measured using a spectrophotometer (UV 1800, Shimadzu, Japan) [34] at 220 nm to find out the amount of drug entrapped in the nanoparticles [35].

The % EE was estimated using the following formula:

(1) $$\%\;\mathrm{EE}=\frac{\mathrm{Amount}\;\mathrm{of}\;\mathrm{drug}\;\mathrm{in}\;\mathrm{nanoparticles}}{\mathrm{Amount}\;\mathrm{of}\;\mathrm{drug}\;\mathrm{used}\;}\times 100\;$$

The percentage of drug loading was estimated using the same procedure followed for EE. Then % drug loading was calculated by using the formula mentioned below.

(2) $$\%\;\mathrm{Drug}\;\mathrm{loading}=\frac{\mathrm{Amount}\;\mathrm{of}\;\mathrm{drug}\;\mathrm{in}\;\mathrm{nanoparticles}}{\mathrm{Amount}\;\mathrm{of}\;\mathrm{nanoparticles}\;\mathrm{formed}\;}\times 100$$

### 2.8. Coating of the Optimized Nanoparticles

Eudragit EPO is soluble in acetone; thus, the emulsion solvent evaporation technique was employed for the coating of prepared nanoparticles [36]. Different core-(nanoparticles)-to-coat ratios were selected to check their effect on drug release. Light liquid paraffin was selected as the external phase and Tween 80 was used as an emulsifier for the coating. Optimized batch D8 was selected as the core and the coating was done by the method described in the literature with minor modifications [37]. Briefly, the required quantity of polymer was dissolved in acetone and previously prepared chitosan nanoparticles were added to the polymeric solution with constant stirring. The mixture was then added dropwise to light liquid paraffin and Tween 80 solution with magnetic stirring at constant speed for 2 h to evaporate acetone completely. The hardened nanoparticles were recovered by centrifugation and washed three times in petroleum ether to remove the excess of oil. Nanoparticles were then lyophilized and stored in a cool and dry place. Different ratios of nanoparticles and the coating polymer-eudragit (1:1 (batch C1), 1:3 (batch C2), and 1:5 (batch C3)) were tested to fabricate the coated nanoparticles.

### 2.9. FTIR Analysis

Compatibility study was carried out using a Jasco FT-IR spectrophotometer (FP-6500, Tokyo, Japan). The IR spectrum of the pure drug (2 mg), chitosan (2 mg), and nanoparticles’ formulation D8 (equivalent to 2 mg of drug) was studied by preparing potassium bromide (KBr) pellets with 98 mg KBr [38]. The prepared pellets were then scanned 50 times over the range of 4000–400 cm^−1^ wavenumber. The characteristic absorption peaks of rivastigmine tartrate and chitosan at different wavenumbers were compared with the peaks obtained in the nanoparticles’ formulation.

### 2.10. In Vitro Release of Rivastigmine from the Coated Nanoparticles

The in vitro release of rivastigmine from the eudragit-coated nanoparticles (batches C1, C2, and C3) and the optimized uncoated nanoparticle (batch D8) were studied similarly to the procedure mentioned in Section 2.6.

### 2.11. In Vitro Drug Permeability Study

In vitro rivastigmine transport from the nanoparticles was studied using the freshly excised sheep nasal mucosa obtained from a local slaughterhouse (Ahmedabad, India). Nasal mucosa was sandwiched between the donor and receptor cells with the mucosal side directed towards the donor cell. The active diffusion area for drug permeation was 1.13 cm^2^. The receiver fluid was filled with simulated nasal fluid (20 mL), stirred with a magnetic bar at 50 rpm, and the temperature was maintained at 37 ± 0.5 °C [39]. Different ratios (1:1, 1:3, 1:5) of coated nanoparticles equivalent to 12 mg of the drug were dispersed in 1 mL of simulated nasal fluid and placed in the donor compartment. For comparison purposes, a drug solution with the same amount of drug was used as a control. Aliquots were withdrawn at various intervals for 24 h and were replaced with an equal volume of fresh medium. Samples were filtered through a 0.2-µm Millex syringe-driven membrane unit, diluted appropriately, and assayed at λmax 220 nm. The flux and enhancement were calculated according to the formula described elsewhere [40,41].

### 2.12. Measurement of Particle Size, Particle Size Distribution, and Zeta Potential

For the measurement of the particle size, size distribution, polydispersity index, and zeta potential of the nanoparticles, a Zeta sizer (Nano ZS90, Malvern Instruments, Malvern, UK) was utilized. Nanoparticles were dispersed in water and the measurement was done at room temperature [42].

### 2.13. In Vitro Mucoadhesion Testing

This analysis was performed to evaluate the mucoadhesive property of the fabricated nanoparticles according to the method described in literature [43]. To perform this test, a freshly cut 2-cm-long piece of sheep nasal mucosa was collected and tied to a glass slide with the help of clips. Accurately weighed 50-mg nanoparticles were placed on the apical surface of the mucosa. The setup was kept in the humidity control chamber (75 ± 5% humidity) for 10 min to allow the anionic mucosa to interact with cationic Eudragit-coated nanoparticles. Then, this slide was hung at an angle (45°) under the burette tip, which allowed the prepared phosphate buffer (pH 7.0) to flow at a rate of 2 mL/min. The number of dry nanoparticles disadhered from the setup was collected on the Whatman filter paper. The collected nanoparticles were weighed and used to calculate the % mucoadhesion using the following formula [43].

% Mucoadhesion = (Amount of nanoparticles adhere to the mucosa)/(Total amount of nanoparticles applied to the mucosa) × 100(3)

### 2.14. Scanning Electron Microscopy (SEM)

The surface morphology of optimized nanoparticles was observed under the scanning electron microscope. For SEM analysis, nanoparticles (coated and uncoated) were suspended in petroleum ether and a small amount of this suspension was put on an aluminum stud. Following dehydration and fixation, the nanoparticles were coated with gold-palladium by using the SEM coating system POLORON E5100 (sputter coater) in a neutral environment and the morphology of the formulated nanoparticles was measured using a Nova Nano SEM-450 (FEI, Hillsboro, OR, USA). The images were recorded at HT-15 kV with a high-voltage electron beam, and a probe current of 3 × 10^−10^ A was passed through it to scan the nanoparticles [44].

### 2.15. Differential Scanning Calorimetry (DSC)

The thermal nature of rivastigmine tartrate, the physical mixture, and rivastigmine-loaded nanoparticles-optimized formulation was studied using the DSC 4000 system (Perkin Elmer, Waltham, MA, USA). A sample (5 mg) of rivastigmine nanoparticles was precisely weighed and hermetically sealed in aluminum pans. Thermograms were captured by heating samples at a constant rate of 10 °C/min from 30–300 °C. An unfilled sealed pan was employed as a reference [45].

### 2.16. Nasal Ciliotoxicity Studies

Nasal ciliotoxicity studies of nanoparticles of rivastigmine were conducted using sheep nasal mucosa by following the ethical guidelines (VSCP/EC/11508/2020/4, dated 24 August 2020). For this study, three sheep nasal mucosal scrapings, namely, A, B, and C, with uniform thickness were collected and mounted on Franz diffusion cells [46]. ‘A’ was treated with 0.5 mL of phosphate buffer as the negative control, ‘B’ with 0.5 mL of selected nanoparticles (batch C1) of rivastigmine for 2 h as a test, and ‘C’ with 0.5 mL of isopropyl alcohol for 2 h as a positive control. After 2 h, the nasal mucosa was rinsed with saline fluid and subjected to histological studies using hematoxylin-eosin staining [47]. The stained slides were examined using a light microscope (under 400× magnification) and the image was captured using a camera attached with the microscope (ZEISS, Axioscope 5, Jena, Germany).

### 2.17. Statistical Analysis

Data were examined using one-way ANOVA, followed by Turkey’s multiple comparison post-test. The statistical differences between values exhibiting *p* < 0.05 were considered significant.

## 3. Results and Discussion

### 3.1. Preliminary Optimization of Process Parameters for Blank Nanoparticle Preparation

When three different phases were taken for the selection of the external phase for the preparation of nanoparticles, a stable emulsion was formed with heavy liquid paraffin, whereas the flakes were formed with the combination of light and heavy paraffin. On the other hand, light liquid paraffin was found to be suitable for the fabrication of nanoparticles. Therefore, it was selected as an external phase for further investigations. When the stirrer was set at top positions, it showed the formation of small and irregular-sized nanoparticles. Alternatively, setting the stirrer at the bottom position resulted in the development of big and spherical nanoparticles. It was shown that when the stirrer was set at the middle position, it produced better and desirable results when compared to the other two positions. Therefore, the middle position of the stirrer was selected for further studies.

### 3.2. Optimization of Preliminary Batches of the Drug-Loaded Nanoparticles

Preliminary studies (with batches P1–P18) were carried out to select the percentage of chitosan, the amount of drug, the volume of span 80, the crosslinking agent, stirrer speed, and crosslinking time by varying one after another and evaluated for % EE, drug release, and mucoadhesion. It was found that the concentration of chitosan (in batches P1, P2, and P3) had a significant effect on % EE and a minor effect on drug release and mucoadhesion. As the concentration of chitosan was increased, % EE was also proportionately increased. It could be attributable to the fact that when the concentration of chitosan was increased in the formulation, viscosity of the solution correspondingly increased, and, finally, the diffusion of the drug became difficult, which prevented drug release from the particles [48]. A similar observation was noted in the drug release profile as well. Initially, faster drug release was observed at a lower concentration. Alternatively, at higher concentrations of chitosan, initially slower drug release was observed followed by faster release. Increasing the concentration of chitosan in the formulation revealed a comparable effect as that noticed in the drug release as well as drug permeability. At lower concentrations, drug permeability was higher, as drug diffusion became easier from the less dense polymeric structure. There was no significant effect found in mucoadhesion but it was shown that mucoadhesion was slightly increased with an increase in the concentration of chitosan. It was observed that % EE, drug release, and mucoadhesion was good in batch P2 (with 3% chitosan) and P3 (with 4% chitosan) but batch P2 had good syringe ability when compared to batch P3. Thus, batch P2 with 3% chitosan was selected for further studies.

On the other hand, it was observed that % EE was significantly affected by the drug-to-polymer ratio. The % EE was inversely proportional to the drug to polymer ratio in the formulation, where 150 mg (batch P4, drug: polymer, 1:2) showed 45.34% EE but 75 mg (batch P6, drug: polymer, 1:4) showed 71.45% EE. In addition, the drug-to-polymer ratio was found to have a significant effect on drug release as well as drug permeability. It was observed that drug release and permeability retarded markedly at a higher drug-to-polymer ratio (1:4). This could be explained by the fact that the slower the rate of swelling of the polymer, the more controlled the drug release from the matrix [49,50]. Based on the results demonstrating higher % EE, controlled drug release, and sustained permeability, batch P6 with 75 mg drug and 1:4 drug-to-polymer ratio was selected for further studies.

Due to the non-ionic and lipophilic nature of Span 80, it tended to form a stable coating over the spherically dispersed droplets of the emulsion. Based on the results drawn from different batches (P7–P9) of formulations, the aggregation of the nanoparticles was observed at a higher concentration of Span 80. Alternatively, including a higher percentage of Span 80 did not significantly affect % EE, drug release, and drug permeability. Therefore, the batch P8 with 2% of Span 80 was selected for further investigations to avoid aggregation between nanoparticles.

The amount of the cross-linking agent in batches P10–P12 revealed significant alteration in % EE. The higher the amount of crosslinking agent (glutaraldehyde; 4 mL), the higher % EE, which might be because of preventing the leaching of drug from the polymeric structure during the washing step of the nanoparticles [51]. It was proven since the drug release from the polymeric matrix was also retarded from nanoparticles with a higher amount of crosslinking agent. Increasing the concentration of the crosslinking agent might have increased the density of the polymer matrix, thereby retarding the release of the drug from these polymeric particles [52]. A similar effect was observed for drug permeability, where the higher amount of crosslinking agent reduced the permeability of the drug. On the other hand, mucoadhesion was found to be decreased with an increased concentration of the crosslinking agent in the formulation. This might be correlated to the decrease in the availability of free cationic groups of chitosan at higher crosslinking [53]. With increasing the amount of crosslinking agent, particle size was decreased but shrinkage of nanoparticles occurred due to higher crosslinking of particles. Therefore, 3% of glutaraldehyde (batch P11) was considered as the optimum concentration required for further studies as it was providing minimum particle size (150–200 nm), maximum % EE (64.32%), reasonable drug release (65.72% within 8 h), and strong mucoadhesion (85%).

Stirrer speed is the most critical factor in the preparation of nanoparticles. Although, in the present research, the stirring speed did not have any significant effect on % EE, the drug release was significantly affected. As the speed was increased (1000 to 2000 rpm), the particle size of the nanoparticles was found to be decreased from 350 nm (batch P13) to 200 nm (batch P15). On the contrary, the surface area of particles increased; thus, a higher drug release (88.56%) was recorded at the higher stirrer speed (2000 rpm). A similar observation was also made for an in vitro drug permeability test. Alternatively, the particle size was found to enlarge at a lower stirrer speed, which would not be suitable for nasal delivery, as reported earlier [54]. Therefore, 1500 rpm was considered ideal.

On the other hand, the crosslinking time during the fabrication of the nanoparticles had a significant effect on % EE. Improper crosslinking of the polymer matrix resulted in incomplete encapsulation of drugs in the polymer matrix. Therefore, the loaded drug was leaked during the washing period of the fabricated nanoparticles. Additionally, the release of drugs from the nanoparticles (batches P16–P18) was also influenced by the duration of crosslinking. The drug release was largely retarded with increased time of crosslinking since a higher degree of crosslinking causes particles to became denser and thereby hinder their release from the polymer matrix [55]. Further, mucoadhesion of the nanoparticles was reduced due to a higher degree of crosslinking with chitosan contributed by a prolonged period of crosslinking [56]. The particle size was also affected by the extent of crosslinking. As the time increased, particle size decreased significantly. Based on the initial optimization of the different process parameters and compositions, the final composition and parameters of the optimized formulation are depicted in Table 2.

### 3.3. Optimization of the Formulation Parameters Using 2^3^ Full Factorial Design

#### 3.3.1. Drug Release from Rivastigmine Nanoparticles

Rivastigmine nanoparticles’ formulation was optimized using three-factor, two-levels statistical design. Responses of dependent variables for designed batches are shown in Table 3. The statistical data showed that the interaction of three independent variables, drug–polymer ratio (A), crosslinking time (B), and stirrer speed (C) on % drug release after 8 h from rivastigmine nanoparticles and % EE, where the model (2^3^ full factorial design) was found to be significant and the significant influence of model terms A, B, and C on % rivastigmine release from the nanoparticles formulation was confirmed by the *p*-values (*p* < 0.05).

A polynomial equation was generated based on the interaction of three model terms on the % release of rivastigmine from the developed formulations (Equation (4)). The positive coefficient value (+2.08) of C in Equation (4) indicates that the increasing % of drug release could be achieved with increasing the level of stirring speed, whereas the negative coefficient of model terms A (−2.75) and B (−3.39) represented decreasing % of drug release with the increasing level of drug–polymer ratio and crosslinking time. This could be correlated to the findings of the preliminary results. The increased crosslinking time and drug–polymer ratio increased the barrier to release the entrapped drug from the polymeric nanoparticles [57].

% drug release = 75.06 − 2.75A − 3.39B + 2.08C + 0.0762AB − 0.0637AC − 0.3087BC(4)

**Figure 1:**
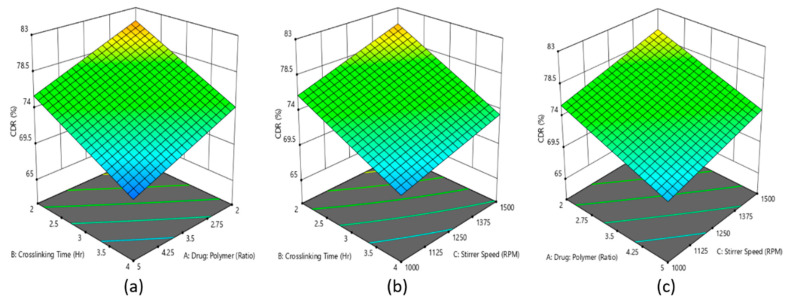
Effect of the interaction of (**a**) drug–polymer ratio, (**b**) crosslinking time, and (**c**) stirring speed is represented in the 3D surface plot on percentage of drug released from rivastigmine nanoparticle formulation.

Further, the similar effect of model terms A, B, and C is reflected in the 3D surface plot (Figure 1a–c), where it is presented that the increasing levels of model terms A led to decreasing % drug release, whereas an increasing level of stirring speed led to increasing % drug release from the fabricated rivastigmine nanoparticles’ formulation.

#### 3.3.2. Entrapment Efficiency of Rivastigmine Nanoparticle

The statistical data indicated that the interaction of three independent variables, drug–polymer ratio (A), crosslinking time (B), and stirrer speed (C) on % EE of the rivastigmine nanoparticles, where the model 2^3^ full factorial design was found to be significant and the significant influence of model terms A, B, and C on % EE of rivastigmine in the nanoparticle formulation was confirmed by the *p*-values (*p* < 0.05).

A polynomial equation was produced based on the interaction of three model terms on % EE of the developed formulations (Equation (5)), where the positive coefficient values for all the three model terms represents the increment of % EE with increasing level of model terms.

% EE = 54.96 + 10.44A + 5.88B − 1.48C + 3.20AB + 0.4043AC − 0.1662BC(5)

**Figure 2:**
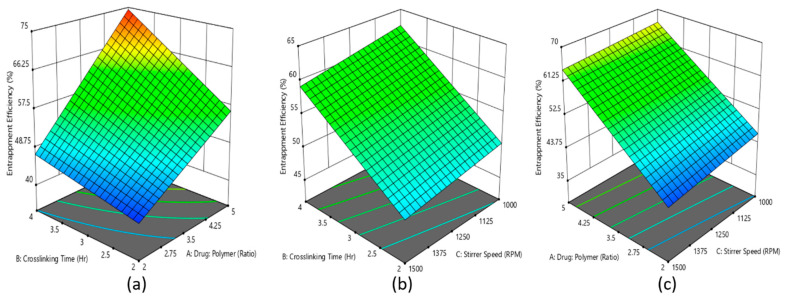
Effect of the interaction of (**a**) drug–polymer ratio, (**b**) crosslinking time, and (**c**) stirrer speed is represented in the 3D surface plot on percentage entrapment efficiency of rivastigmine nanoparticle formulation.

Further, a similar effect of model terms A, B, and C is reflected in the 3D surface plot (Figure 2a–c), where it is presented that the increasing levels of model terms A and B led to increasing % EE of the rivastigmine in nanoparticles.

The 3D surface plot of % EE in Figure 2 signifies that the drug-to-polymer ratio had a more significant effect on % EE when compared with crosslinking time. As the drug-to-polymer ratio increased from 1:2 to 1:5, % EE increased from 40 to 57, while crosslinking time increased from 2 to 4 h and % EE increased from 40 to 48. These results are in good agreement with the % EE existing literature [51]. However, it was observed that the stirrer speed had a negative effect on % EE. Indeed, the increase in stirrer speed led to a decrease in % EE. This is probably because when the stirrer speed was increased, the particles could have broken and help the drug escape into the external phase, which resulted in lower entrapment efficiency.

#### 3.3.3. Checkpoint Batches

**Figure 3:**
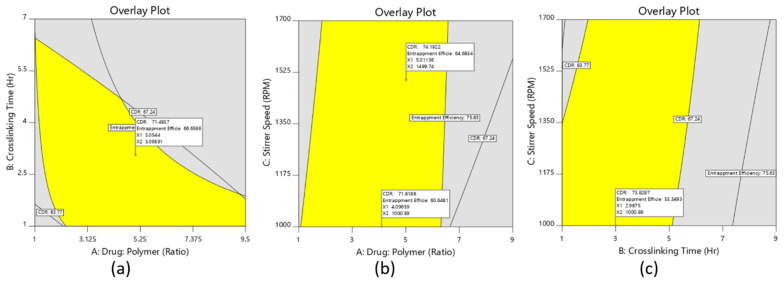
The overlay plots of the nanoparticle formulation, (**a**) overlay plot of the drug-to-polymer ratio and stirrer speed, (**b**) overlay plot of the drug-to-polymer ratio and crosslinking time, (**c**) overlay plot of stirrer speed and crosslinking time.

To identify the design space, the overlay curve was drawn considering percent entrapment efficiency at least above 60–65% and drug release at 8 h below 75%, as shown in Figure 3. Based on the suggestions from the optimization software to validate the optimization process, two checkpoint batches (D9 and D10) were selected from the overlay plots (Figure 3a–c) and the respective analysis was done for those batches. From the result displayed in Table 4, it was observed that practical values of the checkpoint batches were very close to the values of batches that were obtained from the overlay plot. Therefore, it could be concluded that the model used for the interpretation was validated.

From the optimization of different data and validation of the process, the desirability was set to obtain the optimized formulation. Based on desirable characteristics for % EE, particle size, and cumulative drug release (%), the parameters of the optimized nanoparticle formulation are depicted in Table 5, which is also similar to the design batch D8.

### 3.4. Coating of the Optimized Nanoparticles

The drug-loaded optimized chitosan nanoparticle (D8) was then coated with Eudragit EPO to extend the release of nanoparticles to provide 1-day delivery and provide mucoadhesion. Eudragit EPO was chosen as the coating polymer for the chitosan nanoparticles because this polymer is widely used as a coating material in the pharmaceutical field and enhances the mechanical strength and restricts the dissolution rate of chitosan. In addition, it exhibits mucoadhesive properties and has also been used for nasal drug delivery [58,59]. The size of all the coated nanoparticles (batches C1–C3) was found to be <220 nm, with the lowest average size being recorded by batch C1 (~175 nm) followed by batch C2 (~190 nm) and batch C3 (~210 nm). Further, there was no major alteration in % EE from the coated nanoparticles in the formulation, which varied between 64.83% to 69.82% for the core-to-coat ratio of 1:1 to 1:5, respectively.

### 3.5. FTIR Analysis

**Figure 4:**
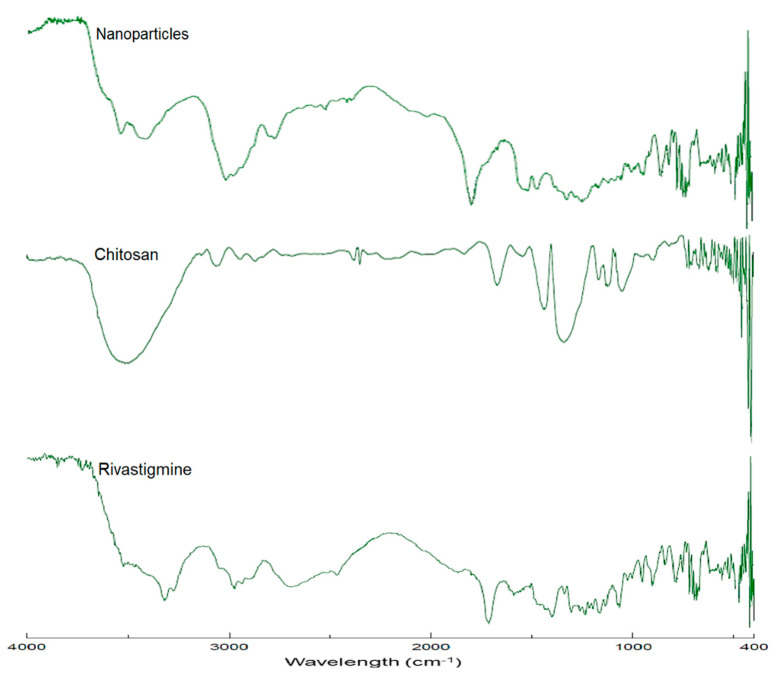
FTIR spectra of rivastigmine tartrate, chitosan, and rivastigmine-loaded nanoparticles (batch D8).

Figure 4 shows the FTIR spectra of pure rivastigmine, chitosan, and drug-loaded nanoparticles (batch D8). The predominant peaks represent the main functional groups of pure rivastigmine that showed characteristic spectral peak positions at 3318 cm^−1^ representing N–H stretching and 1715 cm^−1^ representing C=O stretching (carbamate band); 1401 cm^−1^ refers to C–N stretching in tertiary amines and 954 cm^−1^ corresponds to the =C–H bending. Additionally, there are also other vibrational bands depicted due to the presence of stretching vibrational bands of C=C of the structural aromatic ring, O–H band of the tartrate, and formed N–H between the tartrate and the drug. The observed FTIR spectra of the drug are quite similar to the reported [60]. The spectra of optimized nanoparticles’ formulation also showed all essential peaks of the pure drug with a minor reduction in peak intensity. Additionally, no evidence of the shift in characteristic peaks of the drug was observed. Therefore, it can be confirmed that there were no compatibility issues between the drug and other excipients used in the optimized formulation.

### 3.6. In Vitro Release of Rivastigmine from the Coated Nanoparticles

**Figure 5:**
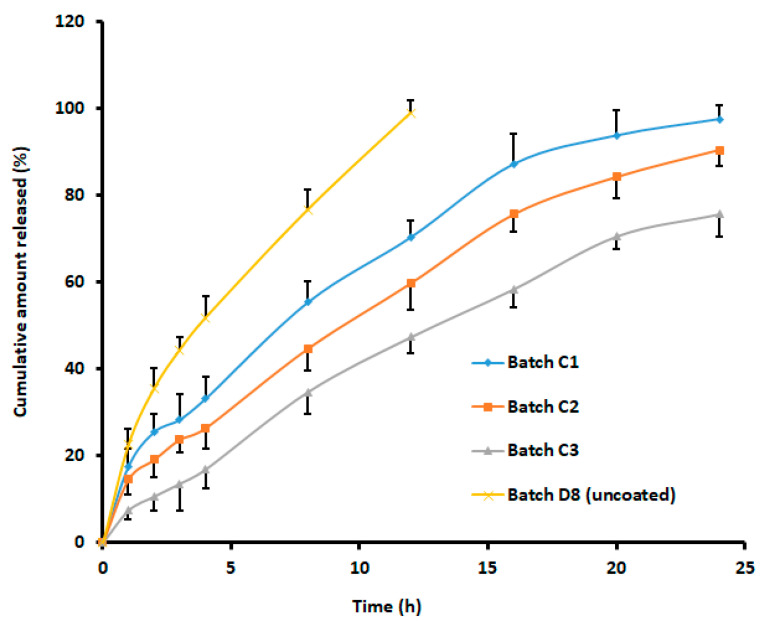
Comparison of cumulative percentage of rivastigmine release from batches with different core-to-coat ratios and control (uncoated). Batch C1 (nanoparticle: eudragit, 1:1), batch C2 (nanoparticle: eudragit, 1:3), and batch C3 (nanoparticle: eudragit, 1:5). The data represent an average ± SD of six trials.

The cumulative release profiles of rivastigmine from the formulated batches of eudragit-coated nanoparticles (batches C1, C2, and C3) and the uncoated batch D8 are presented in Figure 5. Chitosan and its derivatives have been extensively probed as mucoadhesive agents to increase the nasal absorption of hydrophilic drugs and macromolecules due to its ability to decrease mucociliary clearance, enhancing membrane permeability [54], besides interfering with the formation of tight junctions in naso-respiratory epithelial cells [61]. However, results indicated that the cumulative percentage of drug released from rivastigmine loaded in uncoated chitosan nanoparticles using the dialysis sac method was only ~80% in 24 h [16].

Coating with eudragit slowed the release rate of the entrapped drug, which might have been due to an extra barrier created over the chitosan nanoparticles. The cumulative release of rivastigmine from the coated formulations revealed 97.59%, 90.55%, and 75.74% release from the coated formulations with the core-to-coat ratio of 1:1, 1:3, and 1:5, respectively, within the time frame of 24 h (Figure 5). Indeed, this observation could be related to the well-known fact that surface-specific dissolution rate and equilibrium solubility increase with a decrease in particle size. Release kinetics were assessed for batch C1. The goodness of fit models was selected by evaluating r^2^ value, the sum of squares of residuals (SSR), and Fischer Ratio (F) to avoid error in the prediction of the release mechanism. The data indicate a higher r^2^ value (0.990), least SSR value (87.92), and F value (10.99) with the Korsmeyer–Peppas model. The *n* value noticed (0.572) signifies the diffusion mechanism was anomalous transport [62]. Hence, it was concluded that the release of rivastigmine from batch C1 followed the Korsmeyer–Peppas matrix diffusion-controlled mechanism.

### 3.7. In Vitro Permeability of Rivastigmine from the Coated Nanoparticles

**Figure 6:**
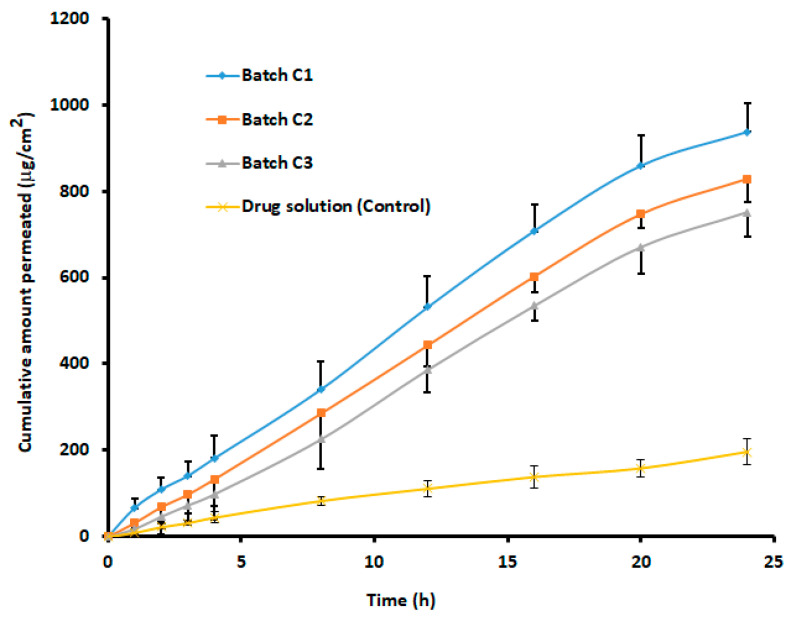
Comparison of rivastigmine permeation across isolated sheep mucosa from batches with different core-to-coat ratios. Batch C1 (nanoparticle: eudragit, 1:1), batch C2 (nanoparticle: eudragit, 1:3), and batch C3 (nanoparticle: eudragit, 1:5). The data represent an average ± SD of six trials.

In vitro release profile does not always correlate with in vivo plasma level-time profile; hence, the suitability of a nasal drug delivery system is generally demonstrated using a permeation study, preferably using sheep nasal mucosa. The permeation of molecules across the biological membrane is generally influenced by the physicochemical properties of the permeant as well as the physiological features of the membrane [63]. The study was conducted for both the coated formulations (1:1, 1: 3, and 1:5) and drug solutions containing equivalent amounts (12 mg) of rivastigmine. Figure 6 exhibits a greater extent of rivastigmine permeation from coated nanoparticles at all ratios through the sheep mucosa in comparison to the drug solution. It was reported earlier that the cumulative percentage of rivastigmine permeated through porcine nasal mucosa from chitosan nanoparticles without any coating was only 70.1% in 24 h [16]. The statistical analysis suggested that the difference in the cumulative amount of drug diffused at 24 h between coated nanoparticles and drug solution was significant (*p* < 0.0001). The steady-state flux (40.39 ± 3.52 μg h/cm^2^) noticed with 1:1 core–coat ratio of nanoparticles was more than 1:3 (36.23 ± 3.97 μg h/cm^2^) and 1:5 (33.19 ± 3.64 μg h/cm^2^) core–coat ratio of nanoparticles. The higher permeation of rivastigmine from the coated nanoparticles demonstrated by flux values could be directly corroborated with the release profile displayed in Figure 5. In addition, the increase in permeation of nanoparticles at 1:1 ratio could be attributed to a minimum particle size that enabled it to penetrate the nasal mucosal layer more efficiently than the larger particle size associated with 1:3 (190 nm) and 1:5 (~200 nm) coated nanoparticles. On the other hand, the lower diffusion of the pure drug solution may have been due to the polar character of rivastigmine, since, for effective transport across nasal mucosa, the drug should have preferably a lipophilic property [64]. From the results, it can be concluded that eudragit-coated chitosan nanoparticles act as an effective drug transporting and targeting carrier because of nano size and unique physicochemical properties.

The in vitro release profile and drug permeability analysis reported from coated formulations with a core-to-coat ratio of 1:1 (batch C1) showed the best results among the three formulations tested. Considering a gradual release and higher drug permeation, these coated nanoparticles (batch C1) with a core-to-coat ratio of 1:1 were selected as an optimized formulation for further characterization.

### 3.8. Particle Size, Particle Size Distribution, and Zeta Potential

**Figure 7:**
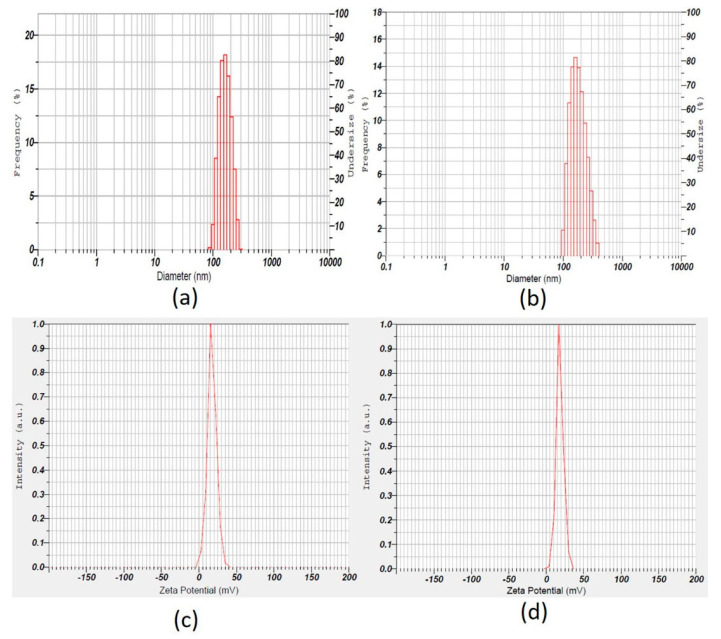
Upper panel: Representative size distribution curve of (**a**) uncoated (batch D8), (**b**) coated nanoparticles (batch C1). Lower panel: Representative zeta potential distribution pictures of (**c**) uncoated (batch D8), (**d**) coated nanoparticles (batch C1).

The particle size and zeta potential distribution of coated and uncoated batches are shown in Table 6 and Figure 7. The designed batch D8 (uncoated) showed nanoparticles with an average particle size of 144.2 ± 28.4 nm (Table 6) and polydispersity index was 0.24 ± 0.03, while batch C1 (coated) showed nanoparticles with an average particle size of 175.4 ± 41.1 nm (Table 6) and a polydispersity index of 0.19 ± 0.03, indicating uniform and narrow size distribution of particles. The zeta potential values exhibited all positive values and greater than 18 ± 3.6 mV for coated and uncoated nanoparticles (Table 6), confirming the stability of nanoparticles and avoiding aggregation behavior [42].

### 3.9. In Vitro Mucoadhesion Testing

To check the adhesion of coated nanoparticles to the nasal mucosa (absorption site) for a prolonged period, in vitro mucoadhesion testing was performed. The results exhibited that all coated nanoparticle batches (C1 to C3) had good mucoadhesive properties ranging from 91–93% and could suitably adhere to sheep nasal mucosa. The results proved that the selected core-to-coat ratio provides higher adhesion probably due to good interaction between the Eudragit^®^ EPO and mucus, as reported early [29].

### 3.10. SEM Analysis of the Nanoparticles

**Figure 8:**
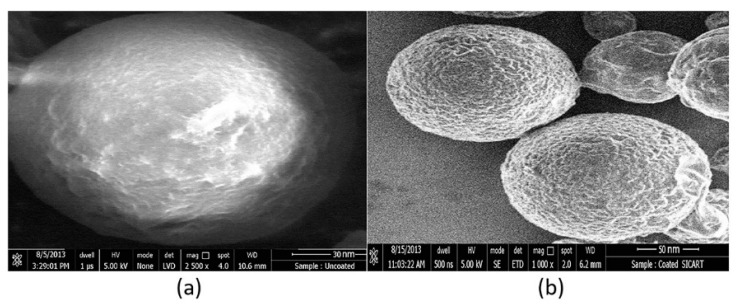
Representative scanning electron pictures of (**a**) uncoated (batch D8), (**b**) coated nanoparticles (batch C1).

The shape and surface of prepared nanoparticles were examined by SEM (Figure 8a,b). From the SEM micrographs, it was noticed that the optimized uncoated nanoparticles’ (batch D8) size was ~145 nm, whereas, upon coating, the size increased slightly from ~145 nm to ~175 nm. Further analysis indicated that all the fabricated coated nanoparticles were less than 200 nm. Our findings are similar to the data reported in the literature [65]. Further, the SEM images in Figure 8 also demonstrate the spherical morphology of the formulated nanoparticles. Furthermore, the image (Figure 8b) indicates that there is no defective coating on the chitosan nanoparticles, which confirms the coating process was done properly on the chitosan nanoparticles.

### 3.11. DSC Analysis of Nanoparticle Formulation

**Figure 9:**
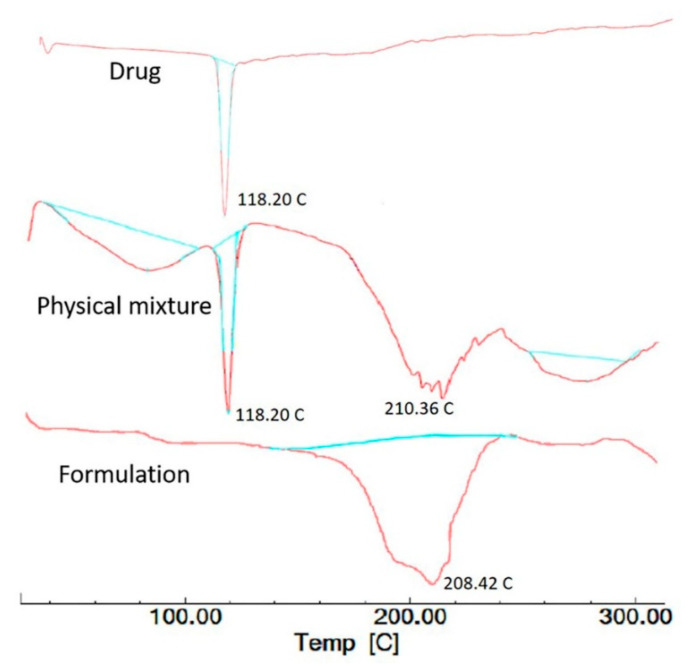
Differential scanning calorimetry patterns of rivastigmine tartrate, physical mixture, and optimized nanoparticle formulation (batch C1).

DSC thermograms of the drug, physical mixture, and nanoparticle formulation (batch C1) are shown in Figure 9. The Thermogram of rivastigmine tartrate shows a sharp endothermic peak at 126.02 °C, confirming the melting point of the drug. The physical mixture shows a sharp endothermic peak of the drug at 126.02 °C and the broad and diminished endothermic peak of chitosan and eudragit EPO in the range of 180–220 °C and 260–300 °C, respectively, which indicates that the drug and polymers were not interacting with each other. However, no characteristic peak of the drug was seen in the thermogram of nanoparticle formulation, which is probably because the drug was encapsulated in the polymeric nanoparticles, as described in the literature [16,66].

### 3.12. Nasal Ciliotoxicity Studies

**Figure 10:**
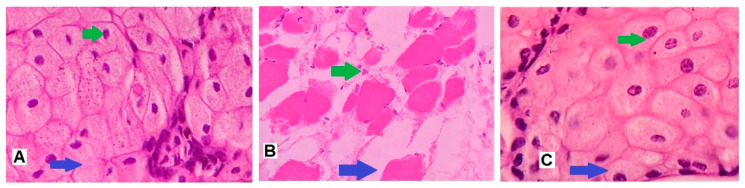
Light microscopic examination of sheep nasal mucosa stained using hematoxylin and eosin stain. (**A**) Negative control: nasal mucosa treated with phosphate buffer shows undamaged nasal cells with unimpaired basement membrane (blue arrow), intact glandular cells with a well-defined nucleus (green arrow). (**B**) Positive control: mucosa treated with isopropyl alcohol illustrates widespread destruction of nasal tissues including the injury to the basement membrane (blue arrow) and glandular cells (with consequent loss of nucleus) (green arrow). (**C**) Test: nasal tissue exposed to rivastigmine drug demonstrates unharmed basement membrane (blue arrow), undamaged glandular cells with distinct nuclei (green arrow).

Figure 10 illustrates the nasal toxicity studies of nanoparticles of rivastigmine (batch C1) performed using sheep nasal mucosa. It is evident from Figure 10 that the sheep nasal mucosal cells treated with phosphate buffer (Figure 10A) and nanoparticles of rivastigmine (Figure 10C) showed intact, undamaged nasal cells with intact basement membrane, suggesting no indication of toxicity of the formulation. However, mucosa treated with isopropyl alcohol (positive control) showed destruction of the normal architecture of the mucosal membrane (Figure 10B). Results of histological studies implied that the formulated rivastigmine nanoparticles are safe to apply to the nasal mucosa, where the formulation will be retained for a longer period due to significant mucoadhesive properties.

## 4. Conclusions

Rivastigmine-loaded chitosan nanoparticles were successfully optimized using pharmaceutical quality design. The preliminary optimization process disclosed the effect of formulation variables, viz. different concentrations of polymer, drug, surfactant, crosslinking agent, and processing variables, such as stirring speed and crosslinking time on the quality of the product. Based on the collected data, the levels of independent variables, i.e., drug–polymer ratio, stirrer speed, and crosslinking time, were set to obtain an optimized batch with spherical nanoparticles with no aggregation, good mucoadhesive property, in vitro release profile, and in vitro permeation. The core-to-polymer ratio (1:1) was fixed to fabricate the coated nanoparticles that control the release pattern of the entrapped drug for a prolonged period. The prepared coated nanoparticles showed size less than 200 nm and released the entrapped drug gradually for 24 h (97.59%) from the 64.83% drug entrapped nanoparticles. Further, in vitro drug permeation through the sheep nasal mucosa revealed greater rivastigmine permeation. Finally, the application of the coated nanoparticles resulted in no toxicity to the nasal cilia of the experimental sheep nasal mucosa. Therefore, developed rivastigmine-nanoparticles could be used as an alternate delivery system to overcome the poor bioavailability issues of the drug. However, this novel drug delivery system should be evaluated further to establish the safety and efficacy in the in vivo experimental models before advancing towards the clinical stage.

## Figures and Tables

**Table 1:** Independent variables with levels and coded values.

Independent Variables			
	Levels		
	+1		−1
A (Drug: Polymer)	1:5		1:2
B (Stirrer Speed (rpm))	1500		1000
C (Crosslinking Time (h))	4		2
Design Matrix with the Independent Variables and Their Coded Values			
Batches	Values of Independent Variables		
	A (Drug: Polymer)	B (Stirrer Speed (rpm))	C (Crosslinking Time (h))
D1	−1	−1	−1
D2	+1	−1	−1
D3	−1	−1	+1
D4	+1	−1	+1
D5	−1	+1	−1
D6	+1	+1	−1
D7	−1	+1	+1
D8	+1	+1	+1

**Table 2:** Formulation composition and characterization parameters of batch P17 to fabricate nanoparticles containing rivastigmine.

Components	Optimized Values
Rivastigmine	75 mg
Chitosan	3%
Span 80	2%
Glutaraldehyde	3 mL
Stirrer speed	1500 rpm
Crosslinking time	3 h
Characterization	
Parameter	Outcome
Average particle size	150 nm
% EE	67.92
% Drug loading	11.98
Aggregation	No
% Mucoadhesion	89
% Cumulative drug release (within 8 h)	82.32

**Table 3:** Responses of dependent variables for designed batches.

Batches	Actual Responses	
	Cumulative Drug Release (%) after 8 h	% EE
D1	78.77	43.46
D2	73.33	57.33
D3	72.55	49.33
D4	67.24	75.63
D5	83.77	40.22
D6	77.90	55.33
D7	76.14	45.06
D8	70.75	73.33

**Table 4:** Predicted and actual cumulative drug release (%) and entrapment efficiency (%) values of the checkpoint batches.

Batches	Drug: Polymer	Crosslinking Time (h)	Stirrer Speed (rpm)	Predicted Value		Actual Value	
				Cumulative Drug Release (%)	% EE	Cumulative Drug Release (%)	% EE
D9	1:5	3	1500	74.19	64.68	71.45	63.34
D10	1:4	3	1000	71.81	60.64	68.76	59.31

**Table 5:** Optimized formulation parameters for the development of rivastigmine nanoparticles (D8) and characterization parameters.

Components	Optimized Parameters
Drug (mg)	60
Chitosan (%)	3
Span 80 (%)	2
Glutaraldehyde (mL)	3
Stirrer speed (rpm)	1500
Crosslinking time (h)	4
Characterization	
Parameter	Outcome
Average particle size	145 nm
% EE	73.33
% Drug loading	11.76
Aggregation	No
% Mucoadhesion	89
% Cumulative drug release (within 8 h)	70.75

**Table 6:** Particle size and zeta potential of rivastigmine nanoparticles.

Batches	Particle Size (nm)	Zeta Potential (mV)
D1	163.2 ± 33.1	22 ± 5.8
D2	166.8 ± 32.8	23 ± 3.1
D3	159.1 ± 29.6	19 ± 4.1
D4	162.4 ± 33.3	25 ± 2.8
D5	154.2 ± 30.7	22 ± 3.7
D6	151.9 ± 28.5	20 ± 4.1
D7	148.9 ± 29.2	20 ± 1.8
D8	144.2 ± 28.4	18 ± 3.6
C1	175.4 ± 41.1	20 ± 2.9
C2	182.3 ± 40.9	23 ± 3.5
C3	192.1 ± 43.9	18 ± 3.1

## Data Availability

All the relevant data are included in the manuscript.
